# The distribution of hepatitis C viral genotypes shifted among chronic hepatitis C patients in Yunnan, China, between 2008–2018

**DOI:** 10.3389/fcimb.2023.1092936

**Published:** 2023-07-11

**Authors:** Yuanyuan Jia, Xiu Zou, Wei Yue, Jin Liu, Ming Yue, Yang Liu, Li Liu, Peng Huang, Yue Feng, Xueshan Xia

**Affiliations:** ^1^ Faculty of Life Science and Technology & The Affiliated Anning First People’s Hospital, Kunming University of Science and Technology, Kunming, China; ^2^ Department of Infectious Disease, Yunnan Provincial Key Laboratory of Clinical Virology, The First People’s Hospital of Yunnan Province, Kunming, China; ^3^ Department of Infectious Diseases, The First Affiliated Hospital of Nanjing Medical University, Nanjing, China; ^4^ Department of Epidemiology, Center for Global Health, School of Public Health, Nanjing Medical University, Nanjing, China

**Keywords:** hepatitis C virus, genotype, RT-PCR, Bayesian analysis, network, transmission

## Abstract

**Object:**

The hepatitis C virus (HCV) is prevalent across China, with a distinctive genotypic distribution that varies by geographical region and mode of transmission. Yunnan is one such geographical region wherein the local population continues to experience a high level of HCV infection, severely straining public health resources. This high prevalence is likely due to the increased incidence of intravenous drug use in that region, as Yunnan is a major point of entry for illegal heroin into China.

**Methods:**

We investigated 510 individuals with chronic HCV infections in Yunnan Province from 2008 through 2018. Using reverse transcription PCR and Sanger sequencing to amplify and sequence samples. Bayesian analyses was performed to estimate the common ancestors and Bayesian skyline plot to estimate the effective viral population size. Molecular network was conducted to explore the characteristics of HCV transmission.

**Results:**

We successfully amplified and sequenced a total of 503 viral samples and genotyped each as either 3b (37.6%), 3a (21.9%), 1b (19.3%), 2a (10.5%), HCV-6 (10.1%), or 1a (0.6%). Over this 11-year period, we observed that the proportion of 3a and 3b subtypes markedly increased and, concomitantly, that the proportion of 1b and 2a subtypes decreased. We also performed Bayesian analyses to estimate the common ancestors of the four major subtypes, 1b, 2a, 3a, and 3b. Finally, we determined that our Bayesian skyline plot and transmission network data correlated well with the changes we observed in the proportions of HCV subtypes over time.

**Conclusions:**

Taken together, our results indicate that the prevalence of HCV 3a and 3b subtypes is rapidly increasing in Yunnan, thus demonstrating a steadily growing public health requirement to implement more stringent preventative and therapeutic measures to curb the spread of the virus.

## 1 Introduction

The hepatitis C virus (HCV) is a global pandemic that continues to rise and constitutes a major threat to public health. The World Health Organization (WHO) has reported that approximately 58 million people have been infected with HCV as of 2022, an estimated 1.5 million new infections occur annually (WHO, 2022), and the prevalence varies among countries. HCV infection can lead to chronic hepatitis C (CHC), cirrhosis, hepatocellular carcinoma, and even death (Calvaruso and Craxi, 2020). Presently, China has the highest HCV disease burden of any single country in the world, and its cases alone account for over 14% of global CHC infections (Li et al., 2019), thus indicating a pressing need for more effective mitigation of the effects of the virus upon the public.

HCV is a single-stranded, positive-sense RNA virus, with a genome of ~9.6 kb in length that encodes a single open reading frame (ORF) flanked by 5′ and 3′ untranslated regions (UTRs). Modern genetic diversity and phylogenetic analyses indicate that the virus is classifiable into eight major genotypes (GTs) and 92 distinct subtypes (Jia et al., 2021). Different HCV GTs have distinct geographical distributions and differ in their responses to antiviral therapies. In general, GTs 1, 2, and 3 are distributed globally, whereas the other five tend to be endemic to certain geographic locales. For example, GT4 is primarily restricted to the Middle East and North Africa, GT5 to South Africa, GT6 to South Asia, GT7 to Central Africa (Chen et al., 2017), and GT8 to India (Borgia et al., 2018). GTs 1, 2, 3, and 6 are the predominant HCV strains found in China today, especially subtypes 1b, 2a, 3a, 3b, and HCV-6 (Zhou et al., 2017).

Yunnan sits on the border of southwest China, adjacent to the so-called “Golden Triangle,” the region where the borders of Myanmar, Laos, and Vietnam meet, well known for its substantial production of opium. Thus, due to this unique geographical circumstance, Yunnan serves as an important drug trafficking route into the mainland of China (Wan et al., 2016). Indeed, historically, Yunnan has been the main channel of heroin entry into China. Unfortunately, HCV transmission and prevalence are critically high in this region and are associated with the migration of the populace and illicit drug trafficking activities. Previous studies have shown that, unlike in mainland China, GT3 was the major GT in Yunnan Province among injection drug users (IDUs), followed by GT6 and GT1, prior to 2008 (Xia et al., 2008). However, research by another group on the Yunnan area from 2009 to 2013 among IDUs found that the distribution of HCV GT subtypes shifted such that HCV-6 was now the predominant GT, followed by GT3b and GT3a (Zhang et al., 2013).

However, little is known about the distribution of HCV GTs across the Yunnan Province. Therefore, we investigated the dynamics of the HCV GT distribution among CHC individuals from 2008 to 2018. Simultaneously, we sought to more comprehensive understand the changes, origins, and spread of the predominant HCV genotypes across Yunnan over time by employing evolutionary and transmission network analyses.

## 2 Materials and methods

### 2.1 Ethical statement

All subjects gave their informed consent for inclusion before they participated in the study. The study was conducted in accordance with the Declaration of Helsinki, and the protocol was approved by the Ethics Committee of Yunnan Provincial Hospital of Infectious Disease (Approval No. YNACC [2015]-12).

### 2.2 Study population

A total of 510 chronic CHC individuals were enrolled in Yunnan Province of China, from January 2008 to December 2018. All the participants provided written informed consent prior to enrollment. Plasma samples were collected from whole blood samples using EDTA tripotassium salt. After centrifugation at 4000 rpm for 10 minutes, the supernatant serum was carefully collected and stored at -80°C for further HCV RNA analysis.

### 2.3 HCV RNA extraction and gene amplification

HCV RNA was extracted from 200-μl serum samples using the MiniBest viral RNA/DNA extraction kit, following the manufacturer’s protocol. The isolated RNA was amplified by nest PCR of *NS5B* region of strain H77 (nt 8266-9303), which was usually used to determine the HCV genotype and subtypes. PCR primers and conditions were reported in previously described studies (Yue et al., 2020).

### 2.4 Sequencing, HCV genotyping and phylogenetic analysis

The HCV *NS5B* gene PCR products were detected using 1.0% agarose gel electrophoresis under UV illumination, purified using a DNA purification kit, and sequenced by Tsingke Biological Technology Co. on an ABI 3730XL automated DNA sequencer. Sequencing date were aligned using Clustal version 1.8.1 and then processed by BioEdit version 7.1.5 software. HCV *NS5B* sequences were genotyped after alignment with reference sequences from the GenBank (available at: http://www.ncbi.nlm.nih.gov/genbank/) according to co-analyses with a total of 111 reference sequences of 1a, 1b, 2a, 3a, 3b, 6a, 6b, 6c, 6d, 6e, 6f, 6g, 6h, 6i, 6j, 6k, 6l, 6m, 6n, 6o, 6p, 6q, 6r, 6s, 6t, 6u, 6v and 6w subtypes. Under the GTR+G+I (general time reversible + gamma distribution + invariant sites) model, Maximum-likelihood (ML) tree was constructed using MEGA version 6.0.6, with bootstrap values estimated as 1000 replications.

### 2.5 Evolutionary analysis

To estimate the epidemic history of the four main HCV subtypes in Yunnan Province, China, four sequence data sets of 1b, 2a, 3a and 3b were assembled. All the data set sequences were from our study, due to few Yunnan sequences had been reported. All the four data sets were aligned and manicured. Then Bayesian coalescent analyses were performed using the Markov chain Monte Carlo (MCMC) algorithm implemented in the BEAST version 1.7.5 under the uncorrelated log-normal relaxed clock model with the GTR+G+I nucleotide substitution model, a coalescent Bayesian skyline plot tree prior, and a relaxed uncorrelated lognormal molecular clock model. For the four subtypes, the evolutionary prior rates were different, and were chosen for their best-fitting by estimating directly from the data sets. For the last, 1.0E-03, 9.0E-04, 9.3E-04 and 2.4E-3 for subtype 1b, 2a, 3a and 3b were used, respectively. Each MCMC analysis was run for 300 million chains, and a tree was output every 10,000 chains. The estimated effective sampling size (ESS) was ≥ 200. Posterior probability densities were determined in Tracer version 1.7.1, and 10% of each chain was discarded as burn-in. The maximum clade credibility (MCC) tree was summarized with Tree Annotator version 1.7 and scanned using FigTree version 1.4.0. In addition, population dynamics were constructed under a coalescent Bayesian skyline plot tree prior and a piecewise linear skyline model with 10 groups using BEAST version 1.7.5. The Bayesian skyline plot was reconstructed using Tracer version 1.7.1.

### 2.6 Transmission network analysis

Based on the four data sets, all sequences were aligned, and pairwise genetic distance were computed using the Tamura-Nei 93 (TN93) method. We performed a sensitivity analysis on the genetic thresholds of subtype 1b, 2a, 3a and 3b separately (Ge et al., 2021). A more conservative (0.001 substitutions/site) to more liberal thresholds (0.05 substitutions/site) were estimated to selected the optimal genetic distance threshold, which was established by the maximum number of clusters below this threshold). We proceeded, visualized and analyzed the identified potential transmission clusters using the Gephi version 0.9.2. A node represents a sequence or an individual, and links (edges) represent connections between different individuals, reflecting their potential transmission relationships, with more links, the node may have higher transmission risk. Pairs were de-fined as connected components of the network comprising 2 nodes, while clusters were more than 2 nodes. Singletons were defined as those greater than the genetic distances threshold sequences. In addition, I roughly divided the sequences into three time periods, 2008~2010, 2011~2014, and 2015~2018, which nodes were rendered with brown, green, and red, respectively.

## 3 Results

### 3.1 Demographic characteristics

This retrospective study included 510 individuals with CHC from Yunnan, China, and spanned 11 years, from 2008 to 2018. The demographic characteristics of the 510 subjects are summarized as follows. The mean age ± standard deviation of participants was 42.5 ± 13.1 years and the majority were male (300, 58.82%). The following clinical characteristics were identified: mean alanine transaminase (ALT), 63.2 ± 58.4 IU/liter; mean aspartate transaminase (AST) 61.8 ± 71.4 IU/liter; and mean HCV RNA, 5.9 ± 2.1 log10 IU/ml.

### 3.2 HCV subtyping and changes across time

**Figure 1:**
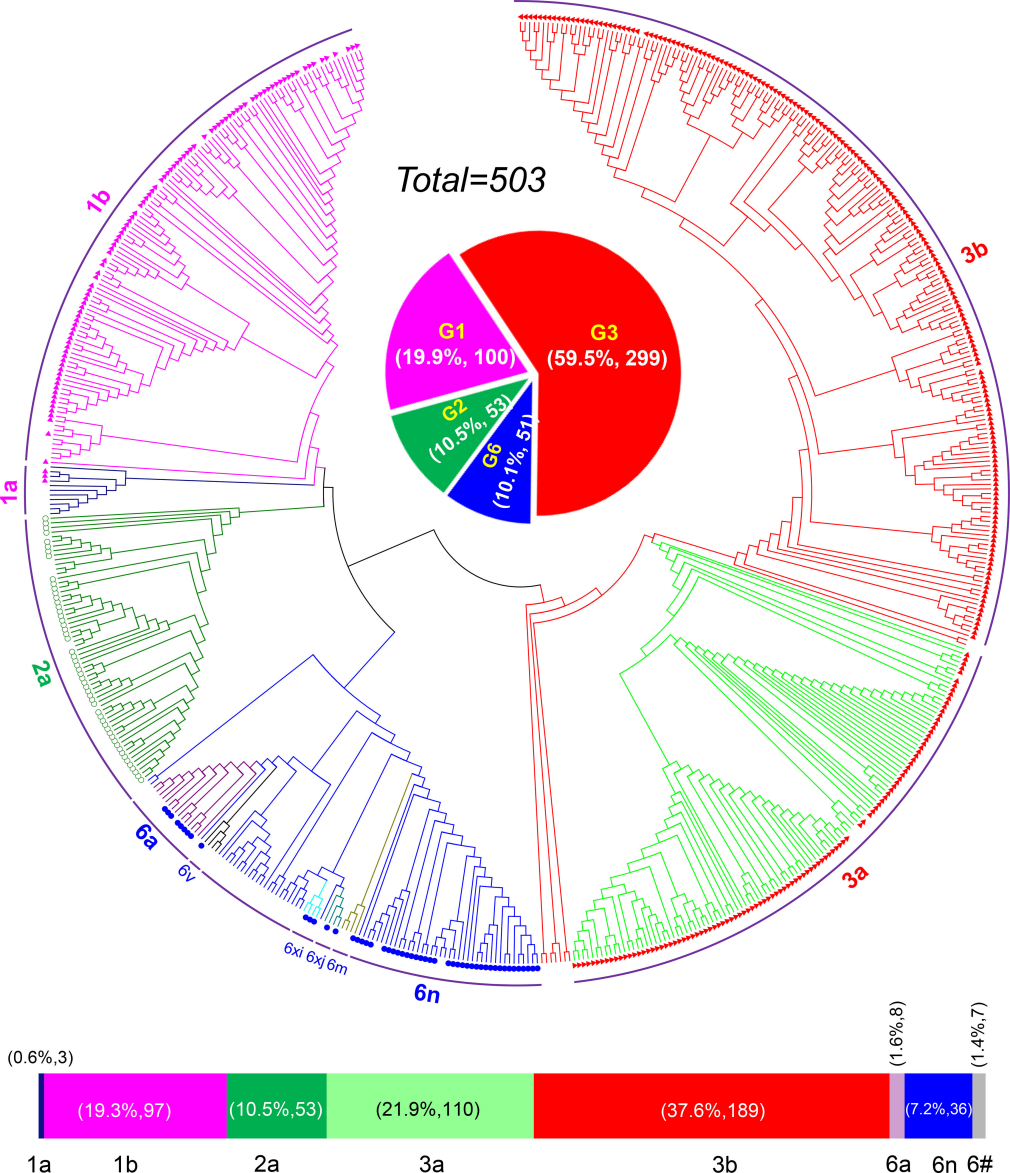
HCV genotyping and prevalence in Yunnan Province from the year of 2008 to 2018.

**Figure 2:**
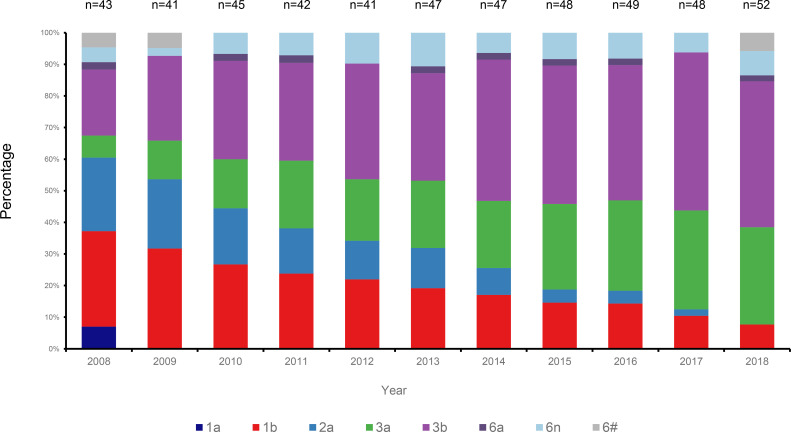
Changes of HCV subtypes over time in Yunnan Province from the year of 2008 to 2018.

HCV genotyping was successfully performed for 503 (98.63%) of the 510 participants. From the 11 years’ worth of sequences, we constructed a circular phylogenetic tree. We found that the most frequent subtypes were 3b (37.6%, 189), then 3a (21.9%, 110), 1b (19.3%, 97), and then 2a (10.5%, 53). Minor identified sequences were mainly GT6 (10.1%, 51), which included 6n (7.2%, 36), 6a (1.6%, 8), other HCV-6 subtypes (1.4%, 7), as well as 1a (0.6% 3) (**Figure 1**). We observed obvious differences in HCV subtype composition during the study period. However, there was no significant difference in the overall number of sequences annually. Interestingly, rather than finding that GT1b was most prevalent, we determined that GT3b was the predominant HCV GT between 2008 and 2018 (GT1b 28.26% to 8.16%; GT3b 19.57% to 48.98%). Simultaneously, the frequency of HCV genotype 3a gradually increased every year (from 6.52% to 32.65%). In contrast, 2a and 1a prevalence decreased from 21.74% to 0 and from 6.52% to 0, respectively. The proportion of GT6 remained stable over time, however, with a lower prevalence but more complexity in its subtypes (**Figure 2**).

### 3.3 Evolutionary and demographic histories of four HCV subtypes

**Figure 3:**
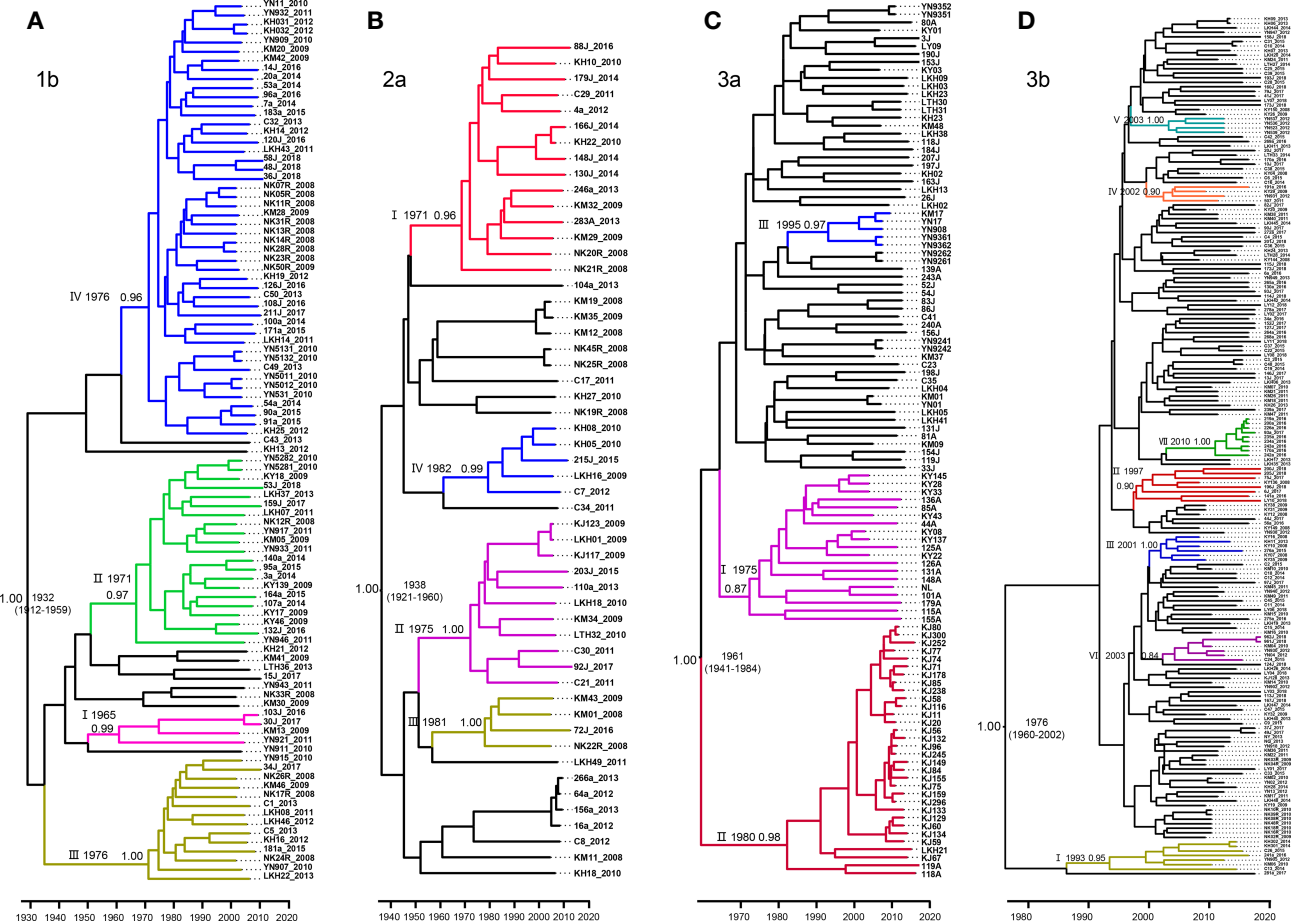
Maximum clade credibility (MCC) tree of the four major HCV subtypes estimated based on partial NS5B gene in Yunnan Province. **(A)** 1b, **(B)** 2a, **(C)** 3a and **(D)** 3b.

MCC trees were constructed for each of our four datasets, each corresponding to a different HCV subtype. From these analyses, we estimated that the common ancestor of the 1b, 2a, 3a, and 3b strains in Yunnan Province date to 1932 (95% highest probability density [HPD], 1912-1959), 1938 (95% HPD:1921-1960), 1961 (95% HPD:1941-1984), and 1976 (95%HPD:1960-2002), respectively (**Figure 3**).

For subtype 1b, we tentatively identified clades I–IV, with posterior probabilities of 0.99, 0.97, 1.00, and 0.96, respectively. CladeI had the earliest scaled divergence time around 1965, followed by cladeII in 1971, and clades III and IV in 1976. Clade IV was the predominant subgroup, with 48 terminal branches (**Figure 3A**).

Among the four data sets, subtype 2a comprised only 53 sequences. Based on the highly supported monophyletic clade, we found four clades of I-IV in 2a with 0.96, 1.00, 0.99, and 1.00 posterior probabilities, respectively. The estimated time of the most recent common ancestor (tMRCA) of clade I was 1971. Clades II-IV dated to 1975, 1981, and 1982, respectively. However, clade I contained the majority, with 15 sequences (**Figure 3B**).

Within the 3a Bayesian tree, three clades (I-III) of closely related sequences with high posterior probabilities were identified. Up to 32 sequences clustered within clade II shared a common ancestor from 1980. Clade II was the second largest with 19 sequences branching out from 1975, and clade III had 5 sequences and branched out from 1995 (**Figure 3C**).

In contrast to the three HCV subtypes above, the Bayesian tree for 3b appeared to be more divergent, with each clade containing only a few sequences. Within this tree, we defined a total of VII well-supported clades with high posterior probabilities ranging from 0.84–1.00, and all emerging within the past 40 years. Estimated tMRCAs were 1993 for clade I, 1997 for clade II, 2001 for clades III, and 2002 for clade IV. Clades V and VI diverged around 2003. And finally, clade VII appeared to diverge as recently as 2010 (**Figure 3D**).

### 3.4 Bayesian Skyline Plot (BSP) analysis

**Figure 4:**
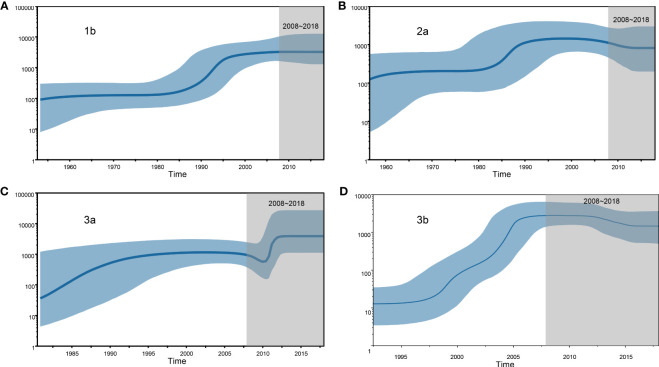
Demographic history of the four HCV subtypes is inferred by Bayesian Skyline Plot (BSP). **(A)** 1b, **(B)** 2a, **(C)** 3a and **(D)** 3b.

We estimated the number of individuals infected with HCV subtypes 1b, 2a, 3a, and 3b by performing BSP analyses using the Yunnan HCV sequences reported in the present study. The estimated viral effective population size (Ne) for subtypes 1b and 2a showed a logarithmic-like growth phase from 1980 to 1995, which was followed by a stabilization of the 1b viral population and a steady decline in the size of the 2a population (**Figures 4A, B**). In contrast, the BSP for the 3a subtype displayed a rapid exponential growth phase from 2009–2012 that leveled off until 2018 (**Figure 4C**). Finally, the 3b subtype BSP showed a clear exponential growth phase from 1995 to 2007 but then tended toward stability over the next 10 years with only a slight decrease in Ne size (**Figure 4D**).

### 3.5 Transmission networks

**Figure 5:**
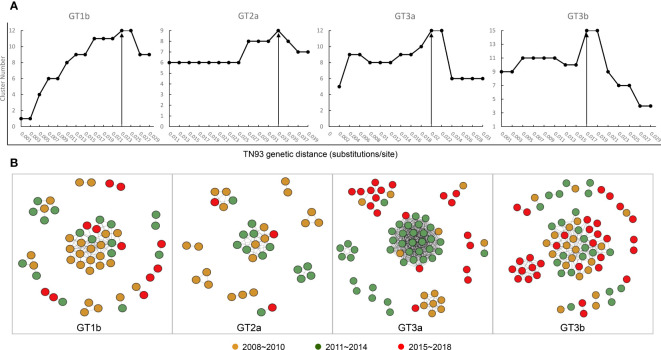
Transmission network definition and visualization. **(A)** Determination of transmission clusters genetic distance through Tamura-Nei 93 (TN93) algorithms. **(B)** Transmission network of the four HCV subtypes shifted between 2008 to 2018.

Next, we constructed transmission networks to model how the HCV strain distribution evolved over time in Yunnan. From this, we identified 48 putative transmission clusters, composed of 234 individuals (52.12%; 234/449). According to the TN93, under the thresholds of 2.3%, 3.3%, 2%, and 1.7% genetic distances of 1b, 2a, 3a, and 3b, respectively, the largest number of clusters was contained in the transmission network. Out of this, 12 clusters were identified among the 54 GT1b sequences, nine among the 36 GT2a sequences, 12 among the 70 GT3a sequences, and 15 among the 74 GT3b sequences (**Figure 5A**). Across the generated networks, the GT3a and GT3b proportions were 18.57% and 27.03% in 2008–2010, 54.29% and 31.08% in 2011–2014, and 27.14% and 41.89% in 2015–2018, respectively. These results clearly indicated that their relative proportions increased rapidly over time. Both GT3a and GT3b sequences were concentrated into large clusters, implying that the 3a and 3b subtypes, particularly GT3b, have spread quickly across the network over recent years. In contrast, the respective proportions of GT1b and GT2a sequences declined over time: 48.15% and 55.56% in 2008–2010, 31.48% and 36.11% in 2011–2014, and 20.37% and 8.33% in 2015–2018, respectively. Interestingly, most of the recent GT1b and GT2a sequences clustered in pairs, indicating a limited ability to spread (**Figure 5B**).

## 4 Discussion

Owing to China bearing the greatest HCV disease burden out of all the countries of the world, a growing number of studies have focused upon characterizing the distribution of the five major HCV subtypes (1b, 2a, 3b, 6a, and 3a) within its borders (Yan et al., 2012; Chen et al., 2017). However, the distribution of HCV GTs and subtypes is not uniform across regions (Zhang et al., 2013). Previous studies have demonstrated that HCV-1b is the predominant HCV GT in most parts of China. However, HCV-2a dominates in northern China. In the south, the genotypic composition tends to be more complex (Chen et al., 2017). GT1b is the major HCV GT in Shanghai, followed by GT3a and finally 3b (Qu et al., 2021). Similar to Shanghai, in Jiangsu the most predominant GTs are 1b, 3a, 3b, and 6a (Qi et al., 2016). Hainan sequences were classified into six GTs: 6a (35%), 1b (31%), 3b (16%), 2a (8%), 3a (6%), and 1a (4%) (Wu et al., 2016). In Guangdong Province, GT1b is the main GT and is followed by GT6a, which replaced 2a as the second most common GT from 2004 onward (Chen et al., 2017). Interestingly, in contrast to the provinces mentioned above, we found the most prevalent GT in Yunnan to be GT3b, followed by GT3a, GT1b, and GT2a, along with various minor GT6 subtypes. Our findings concord well with the results of another recently published study, in which researchers detected 3b isolates in 23 (41.8%) out of 53 donors from Yunnan (Lu et al., 2014) and 24 (30%) from among IDUs (Xia et al., 2008). However, a survey summarizing the changes in the proportions of HCV GTs over time in southwest China before 2017 indicated that GT1b and GT2a decreased while GT3 increased (Zhang et al., 2017). Consistent with the review’s findings, we similarly demonstrated an increased prevalence of 3a and 3b that was accompanied by a decrease in genotypes 1b and 2a in Yunnan. HCV GT3 is associated with a higher risk of liver fibrosis, cirrhosis and cancer than other HCV genotypes (Xu et al., 2022). GT3a and GT3b are the most common subtypes of HCV GT3. GT3a is prevalent worldwide, while GT3b is predominantly found in Southeast and East Asian countries. GT3b has become the second most common subtype in southwest and southern China, including the provinces of Sichuan, Yunnan, Chongqing, Guizhou and Guangdong (Xu et al., 2022). Although the recent discovery of direct-acting antivirals (DAAs) has revolutionized treatment, most patients achieve a sustained virologic response (SVR) of more than 95% (Abulitifu et al., 2022). However, GT3 has a lower SVR than other genotypes, mainly because advanced cirrhosis combined with the presence of resistance-associated substitutions can influence the response to DAA treatment. In China, the DAAs sofosbuvir/velpatasvir or sofosbuvir/ledipasvir are commonly used as treatment regimens for patients with HCV GT3 infection, to which RBV is added for the treatment of patients with GT3 HCV-related compensated cirrhosis and decompensated cirrhosis (Abulitifu et al., 2022). Some studies have shown a significantly lower SVR12 for the GT-3b subtype compared to GT-3a in China, which may be explained by the fact that significant differences in the prevalence of resistance-associated substitutions (RAS) were observed between HCV GT3a and GT3b. More than 90% of subtype 3b HCV strains have baseline RASs at A30K+L31M in the NS5A region (Liu et al., 2022). Worryingly, our results showed a rapidly increasing epidemiological profile of HCV type 3 in Yunnan from 2008 to 2018. Therefore, it is necessary to strengthen the detection of HCV genotypes, subtypes and drug-resistant mutations in Yunnan. In addition, our results showed that HCV GT2a and GT1a decreased from 21.74% to 0 and from 6.52% to 0, respectively. In addition, our results showed that HCV GT2a and GT1a decreased from 21.74% to 0 and from 6.52% to 0, respectively. This may be explained by the fact that treatment of HCV type 2a infected individuals achieved SVR greater than 95% in both the pegylated-interferon-α plus ribavirin and DAA treatment periods (Ishiguro et al., 2015; Li et al., 2022). However, only three cases of HCV GT1a were identified in this study and the change in prevalence is not representative or convincing.

Distinct evolutionary histories have been found for each HCV subtype. In this study, only HCV subtypes 1b, 2a, 3a and 3b were analyzed for evolutionary history because they are the four most common genotypes in Yunnan, while the remaining subtypes 1a, 6a, 6n, etc. were too few in number to be analyzed for evolutionary purposes. We found that the most recent common ancestors of the 1b, 2a, 3a, and 3b strains, as calculated based upon variations in the *NS5B* gene (371 bp), were estimated to be 1932 (95% HPD, 1912-1959), 1938 (95% HPD:1921-1960), 1961 (95% HPD:1941-1984), and 1976 (95%HPD:1960-2002), respectively. We observed a similar divergence time for subtypes 1b, 2a, and 3a in the *E1* region (Wu et al., 2016). It has been shown that in China, the common ancestor of all 1b strains may date to 1942, 2a to 1932, and 3a to 1959. This modest genetic diversity may account for the different gene regions and sequences identified. Notably, there were some differences in subtype 3b. Our results showed that the estimated tMRCAs for all Yunnan strains was approximately 1976. The divergence at 1976 consisted of clade I, or another large branch of the 3b MCC tree using the *E1* gene reported previously, which speculated that 3b isolates originated from Yunnan Province, with the entire tree branching out earlier in 1942 (Wu et al., 2016; Wang et al., 2019). Because published reference sequences for *NS5B* were limited, it is difficult to conduct a reliable overall evolutionary analysis and explore the geographic origins of the four subtypes from China solely using the *NS5B* sequence. However, the present study provides an effective evolutionary rate by performing BEAST analyses of *NS5B* to estimate the dates of strain divergences and thus, effectively characterizes the evolution of the four major subtypes within Yunnan Province.

The history of HCV dissemination varies by mode of transmission. HCV GTs 1 and 2 infections are most likely to be transmitted by blood transfusion, whereas infections of GTs 3 and 6 are associated with having a history of intravenous drug abuse (Wang et al., 2019). To estimate the history of the predominant HCV subtypes in Yunnan, BSP analyses was per-formed separately for each subtype. The results revealed that a phase of rapid population expansion of 1b, 2a, and 3a subtypes occurred during 1980–1995, which may coincide with a period of unsafe blood drawing equipment utilized across China, an incident that led to ~500,000 blood donors being infected with HCV (Shi et al., 1999; Xu et al., 2013). The increase could also be a consequence of the Chinese “open door” policy of the late 1970s that resulted in the in-creased importation of illicit goods (Wang et al., 2019). From 2009 to 2012, 3a showed a rapid exponential phase, which may be associated with the major transmission route of 3a often concentrated on the IDU network via known drug trafficking routes. From 1990 to 2008, transmission via the IDU network and blood transfusions may have accounted for the continued growth in the population size of HCV 3b infections. Alternatively, this increase could also be partially attributable to a growing number of travelers between Yunnan and other Southeast Asian countries. After the rapid growth period of 3b, its BSP curve leveled off before slightly declining. However, several HCV 3b infections have been identified, leading to opportunities to spread into the uninfected population through numerous transmission routes.

Molecular transmission networks have been used to monitor and control emerging outbreaks of HCV. However, it is difficult to identify a unified standard genetic threshold for HCV transmission networks using different genes or even the same gene. Epidemiologically defined outbreaks and epidemiologically unrelated individuals have been re-ported to have a relatedness distance of 3.77% in hypervariable region 1 (HVR1) (Campo et al., 2016). Another publication separately defined *NS5B* (650 bp), *Core-E2* (920 bp), and HVR1 (100 bp) through inter- and intra-person applications of lower and higher cutoffs: 0.018 and 0.020, 0.03 and 0.06, 0.15 and 0.19, respectively, to identify transmission clusters (Olmstead et al., 2015). A recent study used the *Core-E2* (minus HVR1) region of 1221 bp by calculating the TN93 genetic distance to identify the most epidemiologically relevant cut-off, and determined that to be 0.03 substitutions/site for inferring the network (Bartlett et al., 2017). In the present study, we decided to employ the TN93 algorithms, which have been used in HIV infections recently, to perform a sensitivity calculation to measure genetic distances between transmission clusters (Ge et al., 2021). The transmission network results are consistent with our expectations.

Our study does have some limitations. The HCV blood samples collected for this study were provided by the Yunnan Provincial Key Laboratory of Clinical Virology Team, an interferon clinical practice base in Yunnan Province approved by the China Hepatitis Control Foundation, which has 120 sub-centres in Yunnan Province covering 16 prefectures and 120 counties. A random sample of 50 HCV-positive cases was selected each year for this survey, and although the annual random sample was small, the sample in this study is reasonably representative of HCV prevalence in Yunnan Province. To better characterise the epidemiological changes in the distribution of HCV genotypes in Yunnan, it is important to increase the sample size in the future. Another limitation is that sample transmission routes were not obtained, making it difficult to accurately define transmission networks. Finally, the samples we used for BEAST analyses and transmission networks were derived solely from the Yunnan area; therefore, we were unable to analyze the geographic transmission into Yunnan from other regions.

## 5 Conclusions

In conclusion, we evaluated our recent data from 2008–2018 on HCV genotypic distribution dynamics in the Yunnan Province of southern China, which showed that HCV subtypes 3b and 3a had gradually increased to be the predominant GTs of the region, and that 1b and 2a concomitantly decreased. In 2018, 3b became the most predominant sub-type, followed by 3a and then 1b. We performed Bayesian analyses to construct MCC trees for each subtype, which estimated when the common ancestors of 1b, 2a, 3a and 3b, respectively, existed. Furthermore, our BSP and transmission network analyses supported our findings that the distribution of HCV subtype had markedly shifted over time. Overall, our results are of great significance for the epidemiological investigation and prevention of HCV infection, and are especially useful for tracking individuals within transmission clusters.

## Data availability statement

The data presented in the study are deposited in the Genbank repository, accession number OR210427-OR210929. Further inquiries can bedirected to the corresponding authors.

## Ethics statement

All subjects gave their informed consent for inclusion before they participated in the study. The study was conducted in accordance with the Declaration of Helsinki, and the protocol was approved by the Ethics Committee of Yunnan Provincial Hospital of Infectious Disease (Approval No. YNACC [2015]-12). The patients/participants provided their written informed consent to participate in this study.

## Author contributions

YF and XX conceived and designed the experiments. YJ, XZ, JL, and YL performed the experiments and analyzed the data. YJ wrote the original manuscript. WY and LL reviewed and edited the manuscript. WY, MY, and PH provide technical guidance. YF and XX supervision. All authors contributed to the article and approved the submitted version.
